# Protein–Protein Interactions as Promising Molecular Targets for Novel Antimicrobials Aimed at Gram-Negative Bacteria

**DOI:** 10.3390/ijms262210861

**Published:** 2025-11-09

**Authors:** Piotr Maj, Joanna Trylska

**Affiliations:** Centre of New Technologies, University of Warsaw, ul. Banacha 2c, 02-097 Warsaw, Poland; joanna@cent.uw.edu.pl

**Keywords:** Gram-negative bacteria, protein–protein interactions, molecular targets

## Abstract

Antibiotic resistance, especially among Gram-negative bacterial strains, places a massive burden on global healthcare systems as resistance development has outpaced antibiotic discovery. Protein–protein interactions, successful in other therapeutic contexts, are emerging as promising, yet underexplored, targets for the development of novel classes of antibacterials. Pathogen-specific protein–protein interactions are attractive targets because they are often structurally and functionally distinct from host proteins and are less likely to elicit rapid resistance. This review summarizes recent developments in targeting protein–protein interactions in Gram-negative bacteria, focusing on the modulation of five critical cellular processes: membrane regulation, replication, transcription, translation, and toxin-antitoxin systems. We highlight the design and discovery of both small-molecule and peptide-based inhibitors. While many identified modulators exhibit potent in vitro activity against their respective targets, achieving effective penetration of the complex Gram-negative cell envelope remains a major challenge. Nevertheless, the diverse and essential nature of these bacteria-specific protein–protein interactions represents an attractive strategy for developing next-generation antimicrobials to combat drug-resistant pathogens.

## 1. Introduction

Pathogenic bacteria have plagued humanity for thousands of years. Only at the turn of the 20th century did effective antimicrobials begin to emerge. Sahachiro Hata discovered the antisyphilitic properties of salvarsan in 1909 [1]. The first antimicrobial with a relatively broad-spectrum effect, prontosil, was discovered by Gerhard Domagk in 1932 [2]. Crucially, penicillin reached widespread use in the 1940s [3], ushering in the era of antibiotics. Further discoveries followed, initiating the so-called golden age of antibiotic discovery. However, the introduction of new antibiotic classes to the market has effectively stalled after its peak in the 1950s and 1960s [4,5]. Currently, antimicrobial resistance (AMR) presents an urgent crisis in global healthcare systems as we struggle to develop new antibiotics. Numerous bacterial strains are becoming multidrug-resistant (MDR), rendering several classes of antibiotics ineffective. The annual excess healthcare costs of resistant infections in the United States alone have reached $20 billion [6]. Without concerted action, according to the most recent forecasts, by 2050, an estimated 2 million people will die directly from drug-resistant infections each year, with over 8 million additional deaths associated with AMR [7]. New analogs of existing antibiotics are expected to overcome antibiotic resistance only temporarily [8]. Insufficient financial incentives prevent most pharmaceutical companies from investing heavily in the field [9].

Underscoring the gravity of this issue, the World Health Organization (WHO) updated its Bacterial Priority Pathogens List in 2024 [10]. This document is intended to guide research, development, and strategies for preventing and controlling antimicrobial resistance. The WHO list identifies fifteen bacterial pathogens, nine of which are Gram-negative. These strains cause severe infections and lack safe and effective treatment options. They are divided into three priority groups. (1) The critical group comprises pathogens that pose the highest threat to public health due to limited treatment options, high mortality and morbidity, extremely limited means of prevention, and high transmissibility. These pathogens often possess globally disseminated mechanisms of resistance. (2) The high group includes bacterial pathogens that cause a substantial disease burden, have few potential treatments in the development pipeline, and can be critical for specific populations or geographic areas. (3) Pathogens in the medium group present moderate treatment challenges, a moderate disease burden, and relatively more candidates for treatment in the pipeline. Gram-negative pathogens of critical importance are: carbapenem-resistant *Acinetobacter baumanii*, third-generation cephalosporin-resistant Enterobacterales, and carbapenem-resistant Enterobacterales. Fluoroquinolone-resistant *Salmonella* Typhi, fluoroquinolone-resistant *Shigella* spp., carbapenem-resistant *Pseudomonas aeruginosa*, fluoroquinolone-resistant non-typhoidal *Salmonella*, and third-generation cephalosporin- and/or fluoroquinolone-resistant *Neisseria gonorrhoeae* are listed as high-priority pathogens. Ampicillin-resistant *Haemophilus influenzae* belongs to the medium-priority group [10].

Gram-negative bacteria warrant special attention since they include many of the most persistent and widespread pathogens. They are phenotypically and genetically distinct from Gram-positive bacteria, which is most noticeable in the characteristic structure of their cell envelope. Gram-negative bacteria possess an outer membrane, a thin peptidoglycan layer within the periplasmic space, and an inner membrane. Therefore, any antibiotic molecule entering a Gram-negative bacterium faces numerous physicochemical obstacles. After encountering a negatively charged lipopolysaccharide (LPS) layer, the molecule must pass through a hydrophobic outer membrane containing various porins, channels, and receptors, a hydrophilic peptidoglycan layer within the periplasm, and finally another lipid bilayer, the inner membrane [11]. This complex structure, together with the immense genetic plasticity, is largely responsible for the ability of Gram-negative bacteria to develop antibiotic resistance [12].

The factors listed above have not escaped the attention of researchers worldwide, who are proposing novel strategies for developing antibacterials. Among several strategies is the targeting of bacterial protein–protein interactions (PPIs). A significant fraction of proteins expressed by all living organisms interact with other proteins, i.e., form PPIs. Protein complexes are usually formed in a highly selective manner, only between precisely defined protein partners. These complexes play a fundamental role in virtually every biological process, including those underlying genetic, metabolic, and infectious diseases. PPIs range from transient contacts between structurally dissimilar partners to obligatory homooligomerization.

However, until relatively recently, disruption of specific complexes, i.e., blocking PPIs, was not considered a valid therapeutic strategy. The wide, shallow interaction interfaces were perceived as the greatest obstacle in developing PPI inhibitors. Data gathered using structural biology techniques have shown that PPI interfaces can cover an area of approximately 1000–2000 Å^2^ [13], far exceeding the 300–500 Å^2^ typical for enzyme binding sites [14]. This perceived difficulty has since been largely overcome through the identification of hotspot residues. Interaction energy is not uniformly distributed throughout the protein-protein interface; only a small number of hotspot amino acid residues are critical. These hotspot residues tend to cluster in a small area that can often be covered by a short peptide or a small molecule [15], effectively reducing the targetable area to approximately 250–900 Å^2^ [16]. Therefore, PPIs constitute promising molecular targets.

**Figure 1:**
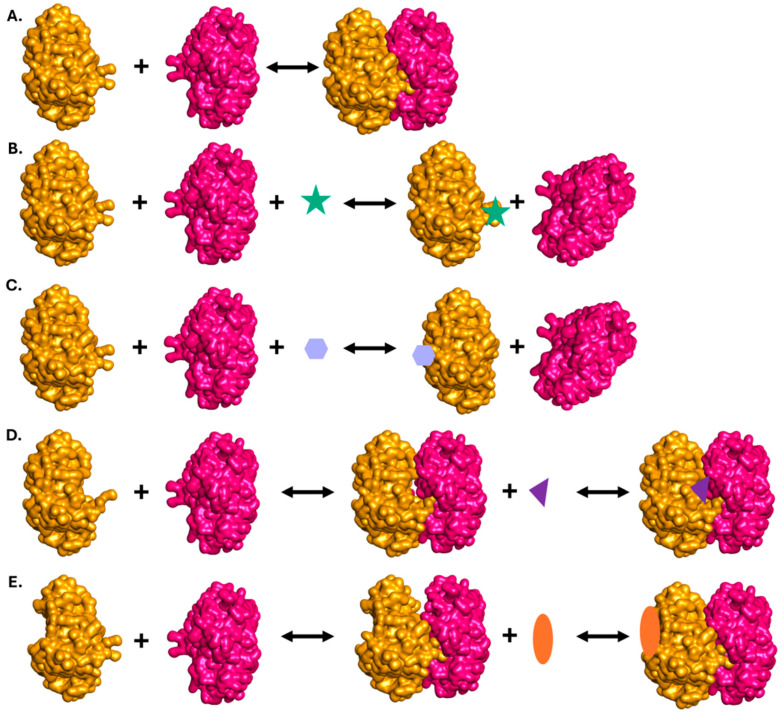
Types of PPI modulators. (**A**). Unmodulated PPI formation. (**B**). Orthosteric inhibitor. (**C**). Allosteric inhibitor. (**D**). Orthosteric stabilizer. (**E**). Allosteric stabilizer.

Due to the large evolutionary distance between humans and bacteria, PPI modulation (inhibition or, in some cases, stabilization) of complexes exclusive to bacteria presents a valid pathway for developing novel antibiotics. This can be achieved through various strategies, depending on the structure and biological function of a particular PPI. A modulating molecule can bind either orthosterically—directly at the interaction interface—or allosterically—at a site distinct from the interaction interface, but in such a way that binding causes conformational changes affecting the PPI [17] (Figure 1).

Clinically relevant PPIs are not limited to bacteria. FDA-approved PPI modulators include, e.g., venetoclax used in the treatment of leukemias, the anti-HIV drug maraviroc, the immunosuppressive agent tocilizumab, and adagrasib and sotorasib for non-small cell lung cancer, among others [17,18,19,20,21,22].

PPIs involving pathogenic bacterial proteins can be divided into two groups: (1) host-pathogen PPIs, in which one interaction partner is eukaryotic (e.g., human) and the other is bacterial, and (2) pathogen-pathogen PPIs, in which both interaction partners are of bacterial origin. This review focuses on pathogen-pathogen PPIs in Gram-negative bacteria that are considered promising molecular targets for developing novel antimicrobial agents. While this topic has been either partially or fully covered in previous excellent reviews [23,24,25,26,27], significant new developments have since emerged, including novel targets and compounds, warranting a comprehensive update and summary. This review will cover the major targetable PPIs in Gram-negative bacteria, organized by their role in key cellular processes: membrane regulation, replication, transcription and translation, and toxin-antitoxin systems.

## 2. Targetable PPIs in Gram-Negative Bacteria

### 2.1. PPIs Associated with Membrane Formation and Regulation

#### 2.1.1. BAM Complex

**Figure 2:**
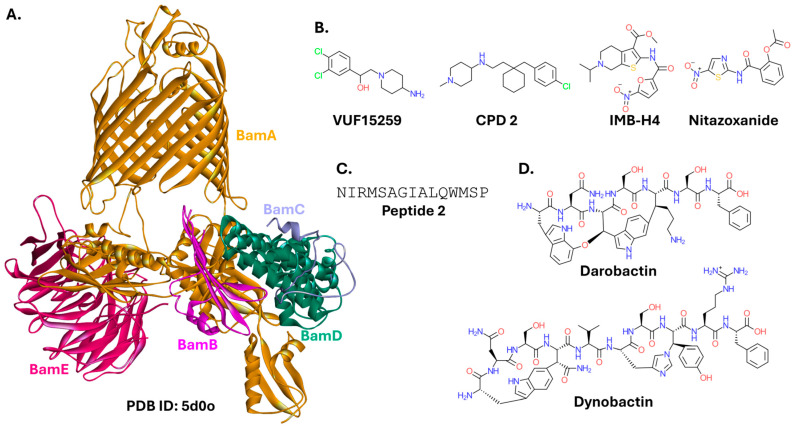
The BAM complex and its modulators. (**A**). Crystal structure of the *E. coli* complex (PDB ID: 5d0o) [36]. (**B**). Structures of small molecules targeting the complex. (**C**). Sequence of a peptide inhibitor. (**D**). Structures of cyclic peptides inhibiting the complex.

The BAM (β-Barrel Assembly Machine) complex, together with associated chaperones, is indispensable for folding and insertion of outer membrane proteins of Gram-negative bacteria. In *Escherichia coli*, the complex is composed of two essential, evolutionarily conserved proteins, BamA and BamD, and the non-essential accessory proteins, BamB, BamC, and BamE (Figure 2A). BAM activity is also dependent on the periplasmic chaperones DegP, Skp, and SurA. BamA consists of a β-barrel embedded in the outer membrane and five periplasmic polypeptide transport-associated (POTRA) domains. BamD and its accessory proteins are all lipoproteins that interact with the BamA POTRA domains to form and stabilize a fully functional BAM complex [28,29]. The BAM complex is conserved among Gram-negative bacteria. However, BamA is also a homolog of the eukaryotic protein Sam50. Sam50 is the key component of the mitochondrial sorting and assembly machinery (SAM complex). Therefore, compounds targeting BamA may be toxic to eukaryotic cells. On the other hand, the remaining members of the BAM complex are not related to SAM, making them potentially more attractive targets for drug development [30,31,32,33,34,35]. While the intricate function and regulation of the BAM complex are only recently becoming apparent, it is garnering attention as a promising target for chemically diverse inhibitors with high antibacterial potential. However, due to this complexity, the exact mode of binding and action of these inhibitors is not always known.

A small molecule containing piperidine and benzene rings (VUF15259; Figure 2B) hampers the production of outer membrane proteins and reduces the levels of the BAM complex. VUF15259 at a 100 µM concentration decreases the growth of *E. coli* strains with a compromised outer membrane protein assembly pathway. However, its exact target and mechanism of action require further investigation [37]. The mechanism of action also remains unclear for two compounds identified in a separate screening campaign: **CPD 2**, a VUF15259 analog containing a cyclohexane ring, and **CPD 14**, containing tetrahydroquinazoline. Their antibacterial activity was also limited. The minimum inhibitory concentration (MIC) against *E. coli* K12 was 50 µM for **CPD 2** and 100 µM for **CPD 14**. The compounds exhibited similar or lower activity against pathogenic strains of *K. pneuomoniae*, *P. aeruginosa*, *A. baumannii*, and *Enterobacter cloacae* [38]. Another research group identified a small molecule with a 4,5,6,7-tetrahydrothieno(2,3-c)pyridine ring system (IMB-H4) that binds to BamA and prevents its interaction with BamD, as confirmed in yeast two-hybrid (Y2H) experiments. This compound inhibits the growth of *E. coli* (MIC = 4 µg/mL against *E. coli* ATCC 25922, and ranged from 8 to 32 µg/mL against different clinical isolates) and several other Gram-negative strains (MICs against *P. aeruginosa* PAO1, *A. baumannii* ATCC 19606, and *K. pneuomoniae* ATCC 700603 were 4 µg/mL, 4 µg/mL, and 32 µg/mL, respectively) [39]. Nitazoxanide, a broad-spectrum antiparasitic and antiviral drug, also inhibits the BAM complex, working in a manner dependent on the presence of BamA, BamB, BamD, and BamE [40].

Peptides have also been developed as BAM complex inhibitors. A 15-residue-long peptide 2 (Figure 2C) derived from the BamA sequence binds to BamD, thereby preventing its interaction with BamA. Expression of a construct containing peptide 2 encoded on a plasmid sensitized *E. coli* cells to vancomycin and rifampicin [41]. Although not a direct PPI modulator, a peptide isolated from *Photorhabdus khanii*, darobactin (Figure 2D), binds at the barrel domain of BamA, inhibiting the activity of the BAM complex. Darobactin was highly active against several Gram-negative strains of *E. coli*, *P. aeruginosa*, *Shigella sonnei*, *K. pneumoniae*, *Salmonella* Typhimurium, and *Moraxella catarrhalis* (MICs of 2–8 µg/mL), and exhibited weaker activity against *Pseudomonas aeruginosa* JMI 1045324, *Enterobacter cloacae* ATCC 13047, and *Proteus mirabilis* KLE 2600 (MICs of 16 µg/mL, 32 µg/mL, and 64 µg/mL, respectively) [42,43,44]. A structurally unrelated peptide with a similar mode of action, but higher efficacy, was named dynobactin. Its discovery resulted from a multidisciplinary approach. Researchers performed a bioinformatic analysis of genes distantly related to the darobactin operon, identified promising candidates in different strains, cultivated them, analyzed products released by bacteria to the medium, and ultimately identified dynobactin produced by *Photorhabdus australis*. Apart from inhibiting the growth of a diverse group of Gram-negative strains (e.g., MIC of 2 µg/mL against *M. catarrhalis* ATCC 25238, 4 µg/mL against *Shigella sonnei* ATCC 25931, and 8 µg/mL against *E. coli* ATCC 25922, *E. coli* K12, *E. coli* AR350, *Shigella flexneri* KLE 2512, *S.* Typhimurium LT2 ATCC 19585, *S.* Enteritidis AR496, *P. aeruginosa* PAO1, *Yersinia pseudotuberculosis* ATCC 6904, and *Vibrio vulnificus* KLE δ-1125; the list of strains was extended compared to ones tested against darobactin), dynobactin showed efficacy in a mouse systemic MDR *E. coli* AR350 infection [45].

Overall, the small molecule, IMB-H4, and cyclic peptides, darobactin and dynobactin, are among the most recent and the most active modulators of the BAM complex. The latter two compounds were identified in symbionts of the nematode gut microbiome, highlighting often-overlooked sources of natural products. Further details on the BAM complex as a molecular target for novel therapeutics are reviewed elsewhere [35,46].

#### 2.1.2. Rcs Complex

The Regulator of the capsule synthesis system (Rcs) is found in members of the order Enterobacterales [47,48]. It is involved in controlling cell membrane integrity under stress. Rcs responds to damage to the outer membrane, including damage to lipopolysaccharides (LPS) and the peptidoglycan network. Rcs is an atypical two-component system (TCS). TCSs are ubiquitous in Gram-negative bacteria but are also found in other organisms and transduce environmental signals. They commonly consist of two elements: a histidine kinase (HK), which undergoes autophosphorylation of a specific histidine residue in response to a signal, and a response regulator (RR) protein, which catalyzes the transfer of the phosphoryl group to the RR receiver domain, thereby affecting downstream processes [49,50]. Rcs is considered atypical because it includes several auxiliary proteins in addition to HK (RcsD) and RR (RcsB) [51,52]. RcsF is a lipoprotein located mainly within the periplasm, anchored by its N-terminus to the outer membrane, and partially exposed to the cell surface. IgaA is an essential inner-membrane protein with periplasmic and cytoplasmic domains [53]. IgaA is a suppressor of the Rcs system and is active under non-stress conditions. During stress, RcsF transiently binds to IgaA, de-repressing the phosphorylation cascade and activating the system [54,55]. Overstimulation of Rcs has a bactericidal effect [54,56]. Of note, proper embedding of RcsF in the outer membrane is mediated by the BAM complex [54,57,58].

**Figure 3:**
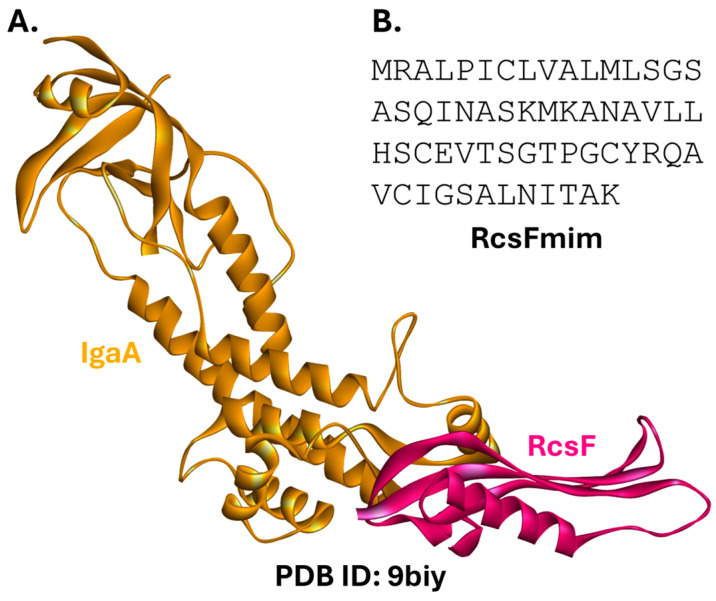
The RcsF-IgaA complex and its modulators. (**A**). Crystal structure of the periplasmic domains of IgaA and RcsF from *E. coli* (PDB ID: 9biy) [59]. (**B**). Sequence of a peptide inhibitor.

Recent structural studies have identified key contact residues for the RcsF-IgaA PPI (Figure 3A). Hotspot residues occupy a small region of the interface, providing a promising site for the design of antibacterials [59]. Indeed, previous protein-protein docking studies led to the initial design of a peptide derived from the RcsF sequence (RcsFmim; Figure 3B) that activates the Rcs complex and slows down the *E. coli* growth rate. However, these effects were observed only when the peptide was overexpressed from a plasmid, necessitating additional research. RcsFmim is also likely to interact with BamA [60].

The RcsF-IgaA PPI remains an unexplored target for small-molecule interventions.

#### 2.1.3. Lpt Complex

**Figure 4:**
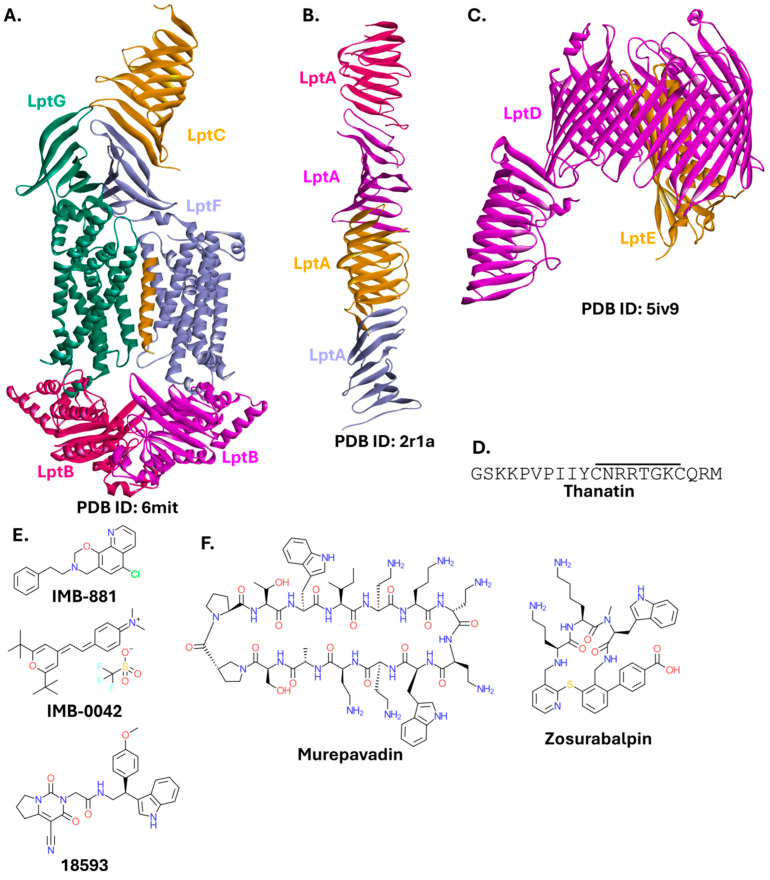
The Lpt complex and its modulators. (**A**). Crystal structure of the LptB_2_FG complex from *Enterobacter cloacae* (PDB ID: 6mit) [64]. (**B**). Crystal structure of the LptA oligomer from *E. coli* (PDB ID: 2r1a) [65]. (**C**). Crystal structure of the LptDE complex from *K. pneumoniae* (PDB ID: 5iv9) [66]. (**D**). Sequence of thanatin. The black line indicates a disulfide bridge. (**E**). Structures of small-molecule inhibitors. (**F**). Structures of cyclic peptidomimetic inhibitors.

The lipopolysaccharide transport (Lpt) complex is a group of seven essential proteins (LptA-G) involved in the translocation of LPS from the cytoplasm to the outer membrane. Lpt is exclusive to Gram-negative bacteria. The heterotetramer LptB_2_FG contains an ATP-binding cassette, utilizing energy from ATP hydrolysis to extract LPS from the outer leaflet of the inner membrane and transport it to the LptC protein. The LptB_2_FGC complex (Figure 4A) then forms, and via the periplasmic protein LptA (Figure 4B), connects with the LptDE complex (Figure 4C) located in the outer membrane. Finally, LPS is released from the LptDE complex through the C-terminal β-barrel domain of LptD [61,62,63].

Due to its crucial role in the physiology of Gram-negative bacteria, the Lpt complex has long been considered a target for new therapeutics. Thanatin (Figure 4D), a peptide derived from insects, blocks the oligomerization of LptA, preventing its interaction with LptC, thereby inactivating the entire Lpt complex [67,68,69]. Compounds structurally dissimilar to thanatin can bind to LptA and effectively block its interaction with LptC, as is the case with the small molecule IMB-881 (Figure 4E), which inhibits the growth of several MDR strains. MICs against different carbapenem-resistant *E. coli* strains ranged from 6.25 to 25 µg/mL. IMB-881 was slightly less potent against MDR clinical isolates of *A. baumannii* (MICs of 12.5–50 µg/mL) and *K. pneumoniae* (MICs of 25–50 µg/mL) [70]. IMB-0042, a compound structurally distinct from IMB-881, can also disrupt the function of the Lpt system. It binds to both LptA and LptC, and inhibits the growth of *E. coli* ATCC 25922 (MIC = 50 µg/mL) [71]. Compound **18593**, identified while screening the Enamine PPI Inhibitor Library and Enamine Diversity Library, exhibits a more stable binding than the previous compounds. However, this was only estimated in silico [72].

Murepavadin is a cyclic peptidomimetic (Figure 4F) that binds to LptD, preventing its interaction with LptA [73,74]. This compound was a promising drug candidate for targeting resistant strains of *Pseudomonas*. Unfortunately, its intravenous formulation was withdrawn from phase III clinical trials due to renal toxicity concerns [75]. Nebulised murepavadin remains in clinical trials [76,77]. Another cyclic peptidomimetic, zosurabalpin, has entered clinical trials as a candidate for the treatment of carbapenem-resistant *A. baumannii* infections. Zosurabalpin targets LptF [78,79].

Despite ongoing research on small-molecule inhibitors of the Lpt complex, cyclic peptidomimetics, murepavadin and zosurabalpin, are among the most promising modulators of any bacterial PPI, with potential to find clinical use. The Lpt complex as a drug target has also been reviewed recently [80].

#### 2.1.4. FimC-FimH

The bacterial adhesin FimH is a protein located at the tip of extracellular filaments called type 1 pili [81]. It is responsible for binding to mannose residues exposed on the surface of host cells [82]. FimH is key to bacterial ability to colonize host cells, constituting a promising target for treating urinary tract infections and Crohn’s disease [83]. Indeed, small molecules [84] and antibodies [85] are being investigated as anti-adhesion drug candidates. This strategy constitutes a textbook example of host-pathogen PPI inhibition. However, the pathogen-pathogen PPI between FimC and FimH can also be targeted. FimC is a periplasmic chaperone essential for the assembly of type 1 pili. It stabilizes individual pilus subunits, including FimH, ensures their proper folding, and prevents their aggregation [86,87]. FimA is the major subunit of type 1 pili, and thousands of its molecules form the bulk of the pilus. FimF regulates the pilus length, and FimG, a minor pilus subunit, also contributes to this regulation. This system is expressed by the members of the *Enterobacteriaceae* family [88].

**Figure 5:**
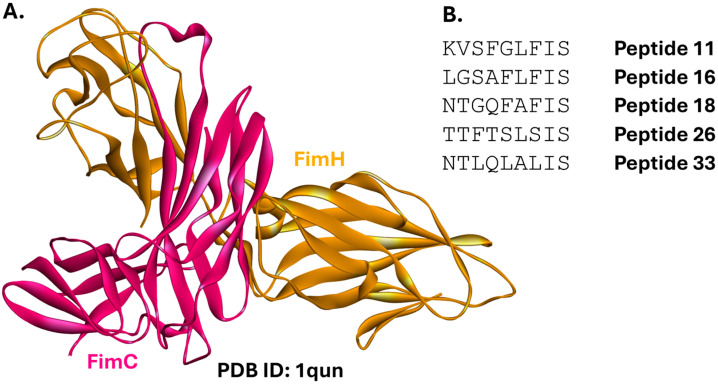
The Fimc-FimH complex and its modulators. (**A**). Crystal structure of the FimC-FimH complex from *E. coli* (PDB ID: 1qun) [89]. (**B**). Sequences of peptide inhibitors.

Structural data have revealed a targetable FimC-FimH PPI (Figure 5A) [89,90]. A nonapeptide library was created based on sequences originating from FimA, FimC, FimF, and FimG (Figure 5B). Several peptides exhibited various levels of FimC-FimH PPI inhibition as assayed by ELISA [91]. Unfortunately, this study has not yet been followed up with investigations into the peptides’ ability to inhibit bacterial growth or suitability of the PPI interface for small-molecule modulation.

### 2.2. PPIs Involved in Bacterial Replication

#### 2.2.1. FtsZ-ZipA

**Figure 6:**
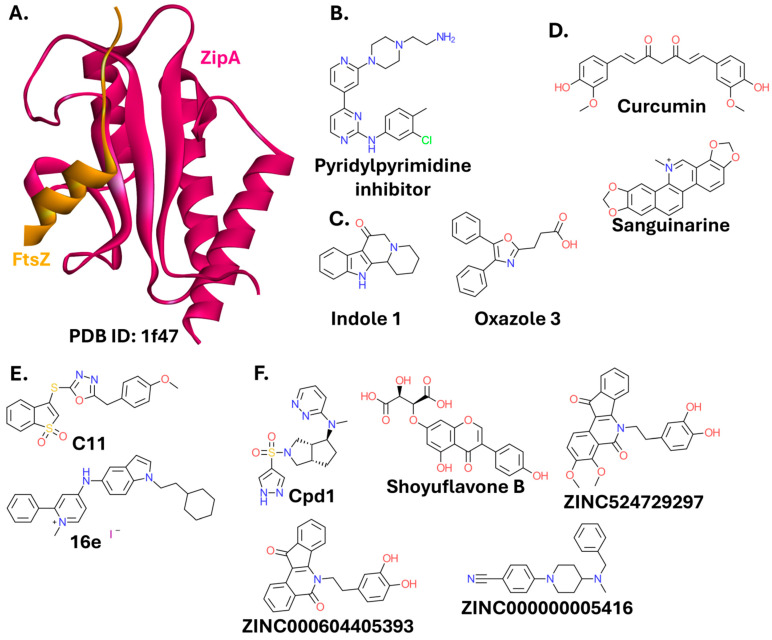
The FtsZ-ZipA complex, its modulators, and compounds targeting the FtsZ assembly. (**A**). Crystal structure of ZipA bound to a fragment of FtsZ from *E. coli* (PDB ID: 1f47) [94]. (**B**). Structure of the pyridylpyrimidine inhibitor. (**C**). Structures of indole and oxazole inhibitors. (**D**). Structures of natural products targeting the FtsZ assembly. (**E**). Structures of synthetic small molecules targeting the FtsZ assembly. (**F**). Structures of computationally predicted small molecules targeting the FtsZ assembly.

Cell division in bacteria relies on the proper assembly of the divisome. The Filamentous temperature-sensitive protein Z (FtsZ), a tubulin homolog, is central to this process. FtsZ polymerizes to form the cytokinetic Z-ring at the cell midpoint. The Z-ring functions as a scaffold for the recruitment of division proteins, which interact to tether and stabilize the ring. ZipA is among the key proteins involved in this process [92]. Unlike FtsZ, which is present in nearly all bacteria, ZipA is expressed by a large subset of Gram-negative strains (*Gammaproteobacteria*) [93,94]. Inhibiting the FtsZ–ZipA interaction (Figure 6A) leads to defective septation and filamentation in bacterial cells. Structural studies have revealed that the FtsZ–ZipA interaction occurs via a 17-mer C-terminal α-helix on FtsZ that binds into a specific pocket on ZipA with micromolar affinity [94,95,96].

A small molecule with a pyridylpyrimidine moiety (Figure 6B) successfully targets the FtsZ-ZipA interaction by binding to ZipA at the PPI interface [97]. However, the molecule is likely highly toxic to eukaryotic cells [98], causing no further exploration of this scaffold. Another screening campaign yielded additional hits containing either indole or oxazole ring systems (Figure 6C), which showed moderate activity against several bacterial strains, and no detected toxicity toward eukaryotic cells. However, the higher activity of these compounds against Gram-positive strains than against *E. coli* suggests they are not entirely specific to the FtsZ-ZipA PPI [99].

FtsZ is also targeted to disrupt its oligomerization or GTPase catalytic activity. Although FtsZ is a homolog of eukaryotic tubulin, this approach is promising, since the two proteins exhibit low sequence identity (below 20%) [100]. This strategy can also lead to broader-spectrum antibiotics, not limited to bacteria expressing ZipA.

Curcumin (Figure 6D), a bioactive compound extracted from turmeric, disrupts the stability of FtsZ polymers. The curcumin binding site on the surface of *E. coli* FtsZ was identified computationally. A homology model of the *E. coli* protein was built on a *Bacillus subtilis* FtsZ template. Binding site analysis and molecular docking revealed a nearly identical binding mode of curcumin within the catalytic core domain of both *E. coli* and *B. subtilis* FtsZ [101]. Sanguinarine, a plant alkaloid, blocks FtsZ assembly in *E. coli* [102]. However, it is toxic to eukaryotic cells due to its binding to the sodium-potassium pump [103] and tubulin [104], halting further research on its direct, antibacterial applications. Virtual screening aimed primarily at the FtsZ of Gram-positive *Staphylococcus aureus* yielded a potent inhibitor, **C11** (Figure 6E), validated in vitro and in vivo. However, this compound also exhibited strong antibacterial activity against Gram-negative strains (*P. aeruginosa* PAO1, *K. pneumoniae* ATCC 13883, and *A. baumannii* ATCC 19606; MIC of 1–16 μg/mL), but only when they were treated simultaneously with an efflux pump inhibitor [105]. Sanguinarine, together with a similar alkaloid, chelerythrine, also served as a starting point for the design of compounds targeting FtsZ oligomerization. Several 1-methyl-2-phenylpyridin-1-ium derivatives were identified. The most potent one, **16e**, inhibited the growth of several Gram-positive and Gram-negative (*E. coli* ATCC 25922, *E. coli* BW25113, *A. baumannii* ATCC 19606, and *K. pneumoniae* ATCC BAA-1902; MIC values of 1–8 μg/mL) strains. Notably, the authors of the study were most interested in targeting bacterial infections threatening animal husbandry and crop cultivation, but the strain selection in antibacterial assays indicates their findings are also applicable to human pathogens. Compound **16e** exhibited high efficacy in a mouse bacteremia model. However, the experiment was limited to mice infected with *S. aureus* ATCC 25923 [106].

Several in silico studies proposing lead compounds for the development of FtsZ inhibitors were published recently. An *A. baumannii* FtsZ inhibitor with an *N*,*N*-dimethylpyridazin-3-amine ring (Figure 6F) derived from the Asinex antibacterial library was identified. Molecular docking results were further evaluated with molecular dynamics simulations [107]. A noteworthy aspect of this study was the use of the WaterSwap method to calculate ligand binding free energy. This approach swaps a water cluster of equal volume and size with the ligand within the binding site of the protein and utilizes a mean value of different calculation methods to estimate the free energy of binding [108,109]. Separate molecular docking studies focused on natural products. Out of 30,000 compounds tested, the molecule designated ZINC14708526 (shoyuflavone B) showed the best binding affinity to *A. baumannii* FtsZ and favorable drug-like properties [110]. In a similar work, two berberine analogs (ZINC524729297 and ZINC000604405393) were suggested as potential leads for *E. coli* FtsZ inhibition. The authors trained a machine learning algorithm on compounds with known IC_50_ values against *E. coli* FtsZ, retrieved from the ChEMBL database. Then, they used the model to evaluate berberine analogs. The compounds identified as active were subjected to docking, molecular dynamics, and free energy of binding calculation [111].

A compound with a piperidine ring (ZINC000000005416) was proposed as a lead candidate for *S.* Typhi FtsZ inhibition [112].

FtsZ assembly was also successfully targeted by peptides. A truncated variant (sequence: GEKLKKIGQKIKNFFQKL) of a cathelin-related antimicrobial peptide (CRAMP) inhibits the growth of *E. coli* (MIC = 20 µM) [113]. Peptides forming disulfide bridges were identified using the phage display technology (peptides designated FtsZp1 and FtsZp2, sequences CSYEKRPMC and CLTKSYTSC, respectively). Although they inhibited the GTPase activity of *P. aeruginosa* FtsZ in vitro [114], they were ineffective in inhibiting bacterial growth. Recently, FtsZp peptides were conjugated to a cell penetrating peptide, (RXR)_4_XB, containing unnatural amino acids, 6-aminohexanoic acid and beta-alanine. The two most potent (RXR)_4_XB-FtsZp conjugates inhibited the growth of *E. coli* BAA 2469, *A. baumannii* ATCC 19606, and *P. aeruginosa* ATCC 27853 with MICs of 12–24 μM [115].

Although disrupting the FtsZ-ZipA complex appears highly promising for the development of selective antibacterials, few studies have targeted this PPI. However, targeting FtsZ oligomerization is an active field of research, yielding potent inhibitors. These include small molecules, e.g., **C11** and **16e**, and peptides, e.g., (RXR)_4_XB-FtsZp.

FtsZ inhibition has been thoroughly reviewed [116] in an article that discusses inhibitors not limited to those targeting the FtsZ-ZipA PPI and Gram-negative bacteria. A more recent review also covers other proteins involved in the formation and function of the bacterial cytoskeleton and divisome [117]. MreB [118,119] and the penicillin-binding protein (PBP) [120] are among the most commonly considered targets.

#### 2.2.2. Single-Stranded DNA-Binding Protein

Single-stranded DNA-binding protein (SSB) serves as an important hub within the DNA replication and repair machinery. SSB binds to single-stranded DNA (ssDNA) and recruits numerous enzymes involved in replication, recombination, and repair by interacting with them usually through a conserved C-terminal sequence (Ct) [121,122,123]. Peptides or small molecules can mimic the Ct motif to bind proteins that interact with SSB and disrupt DNA processing in bacteria. Replication Protein A (RPA) is the primary complex involved in the stabilization and protection of ssDNA in eukaryotes. Since it lacks the Ct motif, RPA interacts with its partner proteins through distinct mechanisms, allowing for selective targeting of PPIs formed by SSB [124,125].

**Figure 7:**
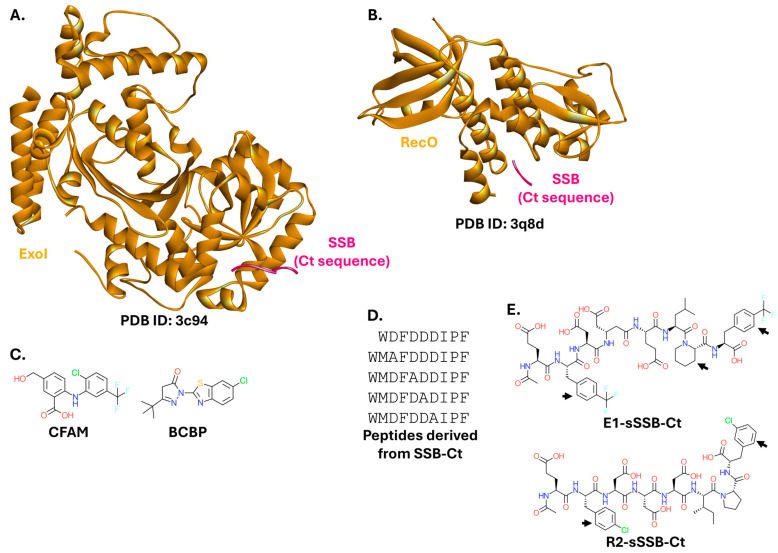
Complexes of SSB with ExoI and RecO, and their modulators. (**A**). Crystal structure of *E. coli* ExoI bound to the Ct sequence of SSB (PDB ID: 3c94) [129]. (**B**). Crystal structure of *E. coli* RecO bound to the Ct sequence of SSB (PDB ID: 3q8d) [134]. (**C**). Structures of small-molecule inhibitors of ExoI. (**D**). Sequences of peptide inhibitors of ExoI. (**E**). Structures of peptide inhibitors containing unnatural amino acids. E1-sSSB-Ct and R2-sSSB-Ct are examples of ExoI and RecO inhibitors, respectively. Black arrows indicate unnatural amino acid residues.

Exonuclease I (ExoI; Figure 7A) and the DNA repair protein RecO (Figure 7B) are among the most commonly targeted SSB interaction partners. ExoI degrades homopolymeric single-stranded DNA but dissociates from double-stranded DNA [126,127]. It is also considered a model for studying SSB-interacting enzymes [128]. Crystallographic studies have revealed a key ExoI binding site for inhibitor design [129]. Small molecules that prevent the SSB-ExoI interaction have been discovered. They bind to ExoI at a site essential for its activity, inhibit the growth of multiple bacterial strains, and are not toxic to eukaryotic cells. The most promising inhibitors were a diaryl compound with a *m*-methoxybenzoate ring (CFAM; IC_50_ = 8 µM for SSB-ExoI disruption and MIC = 36 µg/mL against an outer-membrane-permeable *E. coli imp4213* mutant), and a compound with a benzothiazole ring system (BCBP; IC_50_ = 23 µM and MIC = 62 µg/mL against the same mutant) [130,131] (Figure 7C). ExoI was also targeted and inhibited by peptides derived from the Ct sequence (Figure 7D) [132]. Additionally, peptides modified to include unnatural amino acids exhibit significantly improved affinity for ExoI (E1-sSSB-Ct; IC_50_ = 0.17 µM; Figure 7E) [133]. This approach was also successful for targeting RecO (R2-sSSB-Ct; IC_50_ = 0.59 µM) [133], a recombination mediator protein involved in homologous recombination, replication repair, and DNA annealing in bacteria [134].

**Figure 8:**
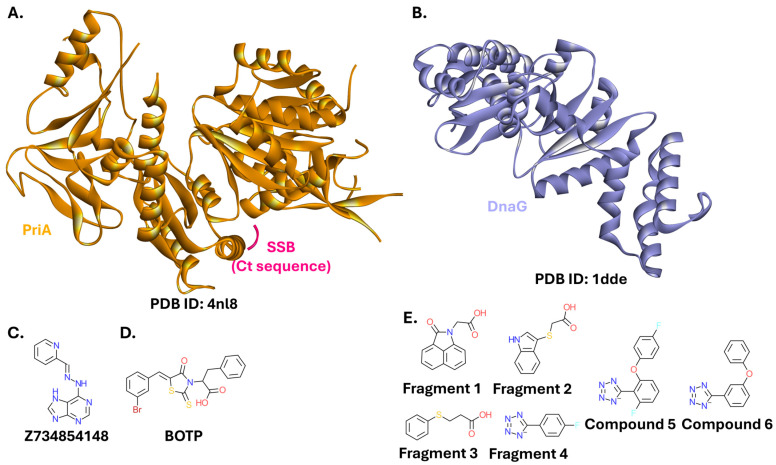
Proteins interacting with SSB, PriA and DnaG, and their modulators. (**A**). Crystal structure of *K. pneumoniae* PriA bound to the Ct sequence of SSB (PDB ID: 4nl8) [141]. (**B**). Crystal structure of *E. coli* DnaG catalytic core (PDB ID: 1dde) [142]. (**C**). Structure of a small-molecule inhibitor of PriA, Z734854148. (**D**). Structure of a small-molecule inhibitor of PriA, BOTP. (**E**). Structures of small-molecule inhibitors of DnaG.

Inhibitors have also targeted other SSB interaction partners. PriA (Figure 8A), one of the proteins responsible for reloading a prematurely dissociated replication complex back onto the replication fork [135,136], was identified as a target of two small-molecule inhibitors in an extensive screening campaign. Seven other compounds also inhibited the SSB-PriA interaction, although no evidence of direct interaction with PriA was found in a differential scanning fluorometry (DSF) assay. However, the structures of these inhibitors were not disclosed. The compounds originated from a commercial small-molecule library purchased from Life Chemicals Inc. (Munich, Germany) [137]. Another project, stemming from a machine learning in silico screening approach, resulted in several hits targeting the SSB-PriA interaction. The most potent (Z734854148, a compound containing a purine heterocycle; Figure 8C) exhibited IC_50_ = 1.3 µM [138]. PriA is also weakly inhibited by BOTP (IC_50_ = 23 µM; Figure 8D), one of the ExoI inhibitors described by Lu et al. [130].

DnaG (Figure 8B) is a primase that produces RNA primers for Okazaki fragments synthesis during DNA replication [139]. A fragment-based screening assay yielded hits binding to this protein, and led to initial efforts to develop them into small-molecule inhibitors (Figure 8E) [140].

Despite multiple studies, inhibitors binding to SSB partner proteins lack high activity against Gram-negative bacteria. However, now scaffolds and chemotypes are emerging. For instance, modified peptides targeting ExoI and RecO are promising antibacterial candidates if equipped with an efficient delivery system.

While not an example of PPI inhibition, SSB binding to DNA has also been inhibited by small molecules, including natural products [143]. Other avenues for targeting DnaG primase, not limited to Gram-negative PPIs, were reviewed by Ilic et al. [144].

#### 2.2.3. β-Sliding Clamp

**Figure 9:**
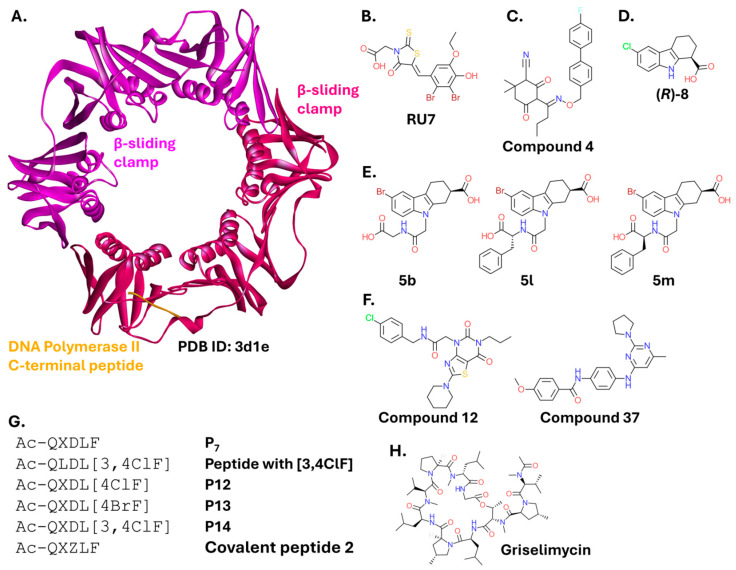
The β-sliding clamp complex and its modulators. (**A**). Crystal structure of *E. coli* β-sliding clamp complex bound to the C-terminal peptide of DNA polymerase II (PDB ID: 3d1e) [148]. (**B**). Structure of a small-molecule PPI inhibitor, RU7. (**C**). Structure of a small-molecule PPI inhibitor, Compound 4. (**D**). Structure of a small-molecule (fragment) PPI inhibitor, (*R*)-8. (**E**). Examples of structures of small-molecule PPI inhibitors designed based on (*R*)-8. (**F**). Examples of structures of small-molecule PPI inhibitors with different scaffolds. (**G**). Sequences of peptide PPI inhibitors (Ac = N-terminal acetyl group; X = cyclohexylalanine; [3,4ClF] = 3,4-dichlorphenylalanine; [4ClF] = 4-chlorophenylalanine; [4BrF] = 4-bromophenylalanine; Z = chloroacetamidealanine). (**H**). Structure of a natural product PPI inhibitor, griselimycin.

Similar to SSB, the β-sliding clamp is a protein that is crucial for DNA replication and repair. It is a ring-shaped protein (Figure 9A) that interacts with multiple partners through a short, conserved clamp binding sequence. Its key interaction partners include DNA polymerases and DNA ligases; thus, disruption of these interactions halts DNA replication, leading to bacterial cell death [145,146]. Although all organisms express proteins with a DNA sliding clamp functionality, eukaryotic homologs of the bacterial β-sliding clamp are highly dissimilar. Therefore, PPIs involving the β-sliding clamp are considered an attractive target for antibacterial drug discovery [147].

A small molecule, RU7 (Figure 9B), inhibits the β-sliding clamp-DNA polymerase interaction, most effectively preventing the binding of DNA polymerase III [148]. Further research led to the discovery of several other chemical scaffolds that bind at the same site. These include a biphenyl oxime compound (Compound **4**; Figure 9C) [149] and a fragment with a tetrahydrocarbazole core ((*R*)-8; Figure 9D) exhibiting good antibacterial activity (with MIC against *E. coli* DH5α and *Acinetobacter baylyi* ADP1 of 78 µg/mL, and even higher activity against Gram-positive strains) [150]. This compound served as the basis for the design of larger compounds (Figure 9E) with improved affinity for the β-sliding clamp in vitro [151]. This work also identified two additional scaffolds, thiazolo [4,5-*d*]-pyrimidinedione and benzanilide (Compound **12**, and Compound **37**, respectively; Figure 9F), that were highly effective at displacing the β-clamp protein partner and inhibiting DNA replication. Compound **12** and Compound **37** inhibited the β-sliding clamp/consensus sequence interaction with IC_50_ values of 15.46 µM and 58.91 µM, respectively. Compound **37** exhibited an MIC of 25 µM against permeabilizer-treated *E. coli* DH10. However, Compound **12** partially inhibited the growth of Gram-positive *Bacillus subtitlis* [152].

Peptides have also been investigated as inhibitors of PPIs that involve the β-clamp (Figure 9G). They were derived from the sequences of the β-clamp’s protein interaction partners [153] and modified by introducing unnatural amino acid residues [149,154]. A different approach to the design of peptide inhibitors of the β-sliding clamp was demonstrated recently. Peptide-based covalent inhibitors targeted a conserved histidine residue in the β-clamp’s peptide-binding pocket. Chloroacetamide-based inhibitors showed high selectivity and greater activity than noncovalent analogs in in vitro inhibition assays (K_D_ = 220 nM) [155]. A long-known natural product, a cyclic peptide called griselimycin (Figure 9H), is a PPI inhibitor targeting the β-sliding clamp. Griselimycin and its synthetic derivatives were initially described as potent only against *Mycobacterium tuberculosis* [156]. However, a recent study demonstrated the high affinity of griselimycin for the β-sliding clamp from multiple Gram-negative strains. In a surface plasmon resonance (SPR) assay, the compound bound to β-clamp orthologs originating from diverse strains including *P. aeruginosa*, *K. pneumoniae*, *Helicobacter pylori*, *Rickettsia typhi*, *Borrelia burgdorferi*, and *Bartonella birtlesii*, with K_D_ values ranging from 7 to 496 nM [157].

Similar to the inhibitors of SSB protein partners, compounds targeting the β-sliding clamp in Gram-negative bacteria are far more effective in vitro than in vivo, due to permeability issues. However, interesting and promising approaches have emerged recently. Compound **12** and Compound **37** represent two chemical scaffolds of small-molecule inhibitors. Pentapeptides can be modified into covalent inhibitors of the bacterial sliding clamp and griselimycin provides another example of a natural product with high antibacterial potential.

The topic of bacterial and eukaryotic (as targets for anticancer chemotherapeutics) DNA sliding clamps in drug discovery has been reviewed elsewhere [147].

### 2.3. Bacterial Transcription Machinery

#### 2.3.1. RNA Polymerase

**Figure 10:**
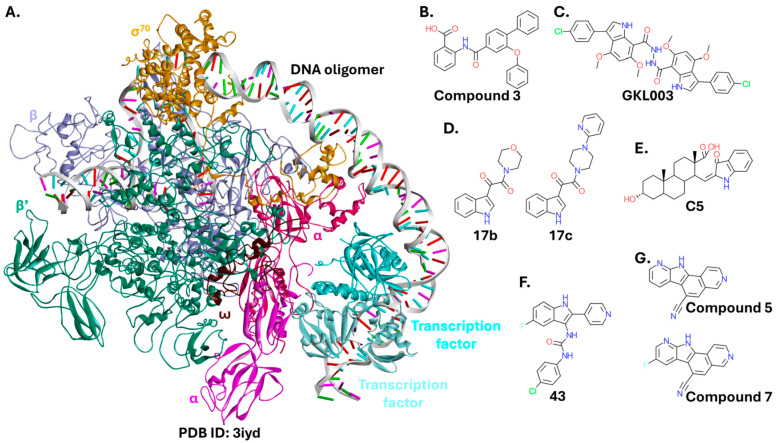
The RNA polymerase (RNAP) complex and its modulators. (**A**). Cryo-EM structure of *E. coli* RNAP holoenzyme bound to a DNA oligomer, and transcription factors (PDB ID: 3iyd) [164]. (**B**). Structure of one of the small-molecule PPI inhibitors, Compound **3**. (**C**). Structure of a small-molecule PPI inhibitor, GKL003. (**D**). Examples of structures of mono-indole PPI inhibitors. (**E**). Structure of a small-molecule PPI inhibitor, C5. (**F**). Structure of a small-molecule PPI inhibitor, 43 (**G**). Structures of two small-molecule PPI inhibitors with pyrido-pyrrolo-isoquinoline scaffolds.

The core apo-enzyme of bacterial RNA polymerase (RNAP) consists of several subunits, forming a large complex (α2ββ′ω). Together with the σ factor, it forms the complete RNAP holo-enzyme (Figure 10A) [158]. The σ factor increases the specificity of RNAP for promoter DNA regions, enabling transcription initiation at the correct sites [159]. The σ factors (designated σ^70^ in Gram-negative bacteria) are evolutionarily conserved among bacteria, yet they lack any close eukaryotic homologs [160]. RNAP has long been considered a target for antibacterials. For example, rifamycins and fidaxomycins target this enzyme and are in clinical use. However, resistance to these antibiotics is growing [161], necessitating a focus on less explored binding sites of RNAP, such as the interface of the RNAP core-σ^70^ PPI. Successful disruption of this interaction results in a critical failure of the bacterial transcription machinery, leading to cells death. Despite the complexity of interactions between the RNAP core and the σ^70^ factor, hotspot residues within the RNAP core are limited to the β and β’ subunits [162,163].

Targeting the RNAP core-σ^70^ PPI led to the identification of small-molecule inhibitors with an anthranilic acid core (e.g., Compound **3**; Figure 10B). However, while they were effective against Gram-positive strains, they did not inhibit the growth of Gram-negative bacteria, with the exception of an *E. coli* mutant with a multidrug efflux system deficiency (MIC = 13 µg/mL for Compound **3**) [165]. Another group proposed a *bis*-indole compound (GKL003; Figure 10C) that binds to the β’ subunit and inhibits bacterial growth, including *E. coli* DH5α. However, significant growth inhibition required a 1 mM concentration [166]. This was later modified into smaller, *mono*-indole molecules (Figure 10D) with less potent activity in vitro, but likely improved cell permeability. These compounds inhibited bacterial growth at a 200 µM concentration [167]. An inhibitor based on a phenanthrene scaffold was also proposed (Figure 10E). It was highly active against Gram-positive strains, but showed limited activity against *E. coli* [168]. A screening campaign of a 34,000-compound library yielded derivatives with an indole scaffold (e.g., Figure 10F), and antibacterial activity against Gram-positive strains and *E. coli*. However, the compounds were effective against the latter only in the presence of an outer membrane permeabilizer (PMBN, a nonbactericidal derivative of polymyxin B) [169]. This pipeline has been recently enhanced in terms of throughput, scalability, and the depth of compound characterization. Screening compounds from three separate libraries resulted in hits with improved potency. Two compounds with pyrido-pyrrolo-isoquinoline scaffolds (Compound **5** and Compound **7**; Figure 10G) inhibited *E. coli* DH10T1R growth with MIC values of 0.78–1.56 µM. However, the MIC values were determined in the presence of PMBN. Compound **5** and Compound **7** did not affect the growth of Gram-positive (*B. subtilis*) and eukaryotic (*Saccharomyces cerevisiae*) cells [170].

Peptides have also been developed to target the interaction between RNAP and σ^70^. Hüsecken et al. designed 16 peptides derived from β, β’, and σ^70^ sequences, ranging from 15 to 25 amino acid residues. Only some of the peptides derived from σ^70^ (sequence: TNRGLQFLDLIQEGNIGLM) effectively inhibited the catalytic activity of RNAP. The sole peptide derived from the sequence of the β subunit had only trace inhibitory activity against the RNAP core. Moreover, these peptides did not affect the growth of *E. coli* [171]. Subsequent efforts to optimize the most promising peptide through increasing its helicity, using copper-catalyzed azide-alkyne cycloaddition, were unsuccessful. Such stapled peptides lost all RNAP inhibitory activity, which may be due to an overly stabilized structure or added bulkiness [172]. Since then, the focus on the RNAP core-σ^70^ PPI has largely shifted toward small molecules.

Diverging slightly from the interactions between the RNAP core and the σ^70^ factor, Zheng et al. studied the PPI formed between RNAP and the transcription factor, NusG (cf. the next section), which also interacts predominantly with β’, similarly to σ factors. Following virtual screening, several small molecules with two aryl groups joined with a linker were assayed against multiple strains. Some of them exhibited low to moderate activity against Gram-negative bacteria including *A. baumannii* ATCC 19606, *Enterobacter cloacae* ATCC 13047, and *E. coli* ATCC 25922, with MIC reaching 16 µg/mL for the most potent compounds. None of the compounds inhibited the growth of *P.* aeruginosa ATCC 27853. However, the screened compounds were overall more potent against Gram-positive strains [173].

Compound **5** and Compound **7**, identified by Caputo et al., are currently among the strongest inhibitors of the β’–σ^70^ PPI in *E.coli*. However, they require the addition of a membrane permeabilizer to inhibit bacterial growth. A recent review focuses on small-molecule inhibitors targeting the PPIs within RNAP in both Gram-negative and Gram-positive bacteria [174].

#### 2.3.2. N-Utilization Substances NusB and NusE

**Figure 11:**
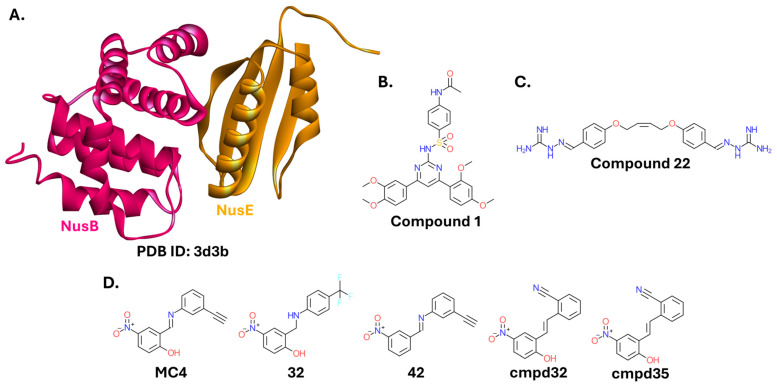
The NusB-NusE complex and its modulators. (**A**). Crystal structure of NusB bound to NusE from *E. coli* (PDB ID: 3d3b) [182]. (**B**). Structure of a small-molecule inhibitor, Compound **1**. (**C**). Structure of a small-molecule inhibitor, Compound **22**. (**D**). Example structures of nusbiarylins.

N-utilization substances (Nus) are transcription factors widely conserved among bacteria. NusA, NusB, NusE (ribosomal protein S10), and NusG, together with the most recently discovered SuhB, form the complete Nus complex, crucial for rRNA expression and folding [175,176,177]. NusB and NusE form a smaller complex that binds to the BoxA sequence, constituting a key step in rRNA transcription [178,179]. NusB-bound NusE can interact with RNAP via NusG [179]. Given its conservation among bacterial pathogens, absence in eukaryotes, and indispensable role in bacterial viability, disrupting the NusB-NusE PPI (Figure 11A) represents an attractive strategy to impair transcription without affecting host cellular processes [180,181].

The screening campaign described by Cossar et al. resulted in promising inhibitors of the NusB-NusE PPI with diverse chemical scaffolds. A pharmacophore design yielded a compound with a pyrimidine ring at its core (Figure 11B) that appeared most successful at inhibiting the growth of *E. coli* DH5α (21% inhibition at a 200 µM concentration) [180]. The same group extended the campaign, focusing on other compounds. A symmetric molecule with two iminoguanidine moieties (Figure 11C) exhibited high activity as a NusB-NusE PPI inhibitor when assayed in vitro, and inhibited the growth of the Gram-negative strains *P. aeruginosa* PA14 and *A. baumannii* ATCC 19606 (MIC ≤ 51 μg/mL) [181]. Notably, the authors initially tested NusB-NusE PPI inhibition with a peptide derived from the NusE sequence (YDHRLLDQS) [180].

Another group conducted a separate screening campaign. The most promising compound, a nitrophenol analog designated MC4 (Figure 11D), was confirmed to bind specifically to NusB, but not NusE, and inhibited the growth of several bacterial strains. Although it was more active against Gram-positive strains, it also exhibited moderate activity against *A. baumannii* ATCC 19606, and *Proteus vulgaris* ATCC 6380 (MICs of 256 μg/mL and 128 μg/mL, respectively) [183]. Since the compound was not toxic to mammalian cell lines, it warranted follow-up studies and exploration of the structure-activity relationship. Further diaryl derivatives were synthesized and tested, resulting in compounds with greatly improved activity against Gram-positive strains, but still with only limited activity against *A. baumannii* ATCC 19606 (most compounds had MICs of 128 μg/mL or higher; only a limited number of compounds exhibited stronger activity). Activity against *A. baumannii* ATCC 19606 was not necessarily correlated with the activity against Gram-positive strains [184]. The entire series of compounds was later collectively referred to as “nusbiarylins” to indicate their function (targeting the NusB protein) and their biaryl structure, where one of the rings is typically being a *p*-nitrophenol. The research initially focused on Gram-positive strains, but two derivatives exhibited moderate to good activity against *A. baumannii* ATCC 19606 (cmpd32; MIC = 32 µg/mL), and *Enterobacter cloacae* ATCC 13047 (cmpd35; MIC = 16 µg/mL) [185]. Subsequent articles from the Cong Ma group, devoted to further developing nusbiarylins, either focused solely on Gram-positive *S. aureus* [186] or did not yield derivatives with improved activity against Gram-negative bacteria [187]. Most recently, they employed Quantitative Structure-Activity Relationship (QSAR) models to explore other potential NusB-NusE PPI inhibitors, diverging from the nusbiarylin scaffold. The authors used the molecules from their previous publications, for which they had activity data against *S. aureus*, to construct a pharmacophore using the Phase module of Maestro. They then generated a 3D QSAR model capable of visualizing how structures affect the antimicrobial activity, and subsequently predicting the activity of hits obtained in virtual screening. Predictions of the 3D QSAR model were validated using a machine learning-based AutoQSAR model, with 70% of molecules assigned to the training set. The pharmacophore model was used to screen a commercial compound library from ChemDiv, with hit compounds subjected to QSAR prediction. Compounds with promising predicted MIC values and beneficial pharmacokinetics were selected and docked to the NusB structure. This pipeline yielded four structurally diverse hits [188]. Although they still require experimental validation, the described pipeline can contribute toward the optimization of modulators of other PPIs.

The NusB-NusE PPI inhibitors are also reviewed elsewhere [174].

### 2.4. Bacterial Translation Machinery

#### L10-L12 PPI

**Figure 12:**
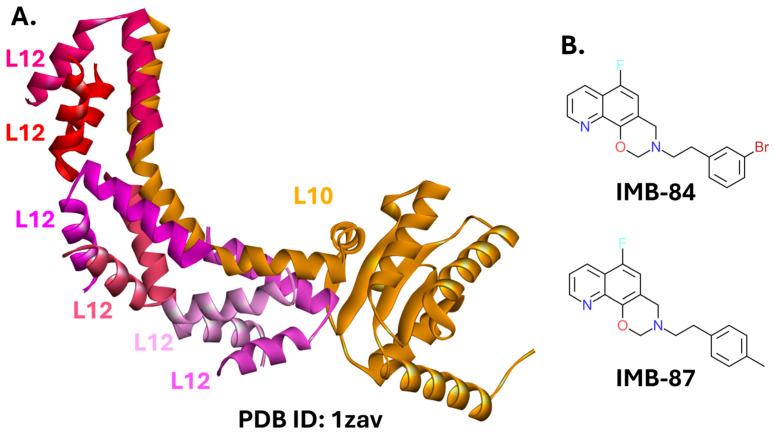
The L10-L12 complex and its modulators. (**A**). Crystal structure of the L10-L12 complex from *Thermotoga maritima* (PDB ID: 1zav) [193]. (**B**). Structures of small-molecule inhibitors.

The interaction between the ribosomal protein L10 (encoded by the *rplL* gene) and multiple copies of L12 (encoded by the *rplJ* gene) (Figure 12A) within the stalk of the 50S ribosomal subunit is essential for the recruitment of elongation factors, making it crucial for ribosomal function and protein synthesis. Elongation factors EF-G and EF-Tu are recruited to the stalk by the L12 C-terminal domain [189]. In the absence of L12, the ribosomal stalk is incapable of interacting with these elongation factors [190]. The L10 protein anchors L12; therefore, disruption of the L12-L10 interaction prevents the binding of elongation factors to the stalk and causes loss of ribosomal GTPase activity [191]. The interaction between L12 and L10 is evolutionarily conserved in Gram-negative bacteria, but these two proteins show low sequence similarity to corresponding human proteins, making this PPI an attractive target for novel therapeutics.

Two small-molecule compounds inhibiting the L10–L12 interaction were identified. Compounds IMB-84 and IMB-87, both with 3,4-dihydro-2H-[1,3]oxazino [5,6-h]quinoline ring systems (Figure 12B), prevent the interaction in vitro and inhibit the growth of *E. coli.* The MICs of IMB-84 and IMB-87 against *E. coli* ATCC 25922 were 2 and 4 µg/mL, respectively. Against eight drug-resistant clinical isolates of *E. coli*, the MICs ranged from 8 to 64 µg/mL. Interestingly, SPR assays indicate that both compounds can bind to either L10 or L12 [192].

### 2.5. Toxin-Antitoxin Systems

Toxin-antitoxin (TA) systems are diverse genetic modules widespread in bacteria. They express a toxin that can kill its parent cell and an antitoxin that neutralizes the toxin’s activity. Several factors, including environmental stress, can disrupt the toxin/antitoxin balance in the cell, allowing the toxin to exert its lethal effect. TAs are currently categorized into eight types that operate through different molecular mechanisms. In type II TAs, both the toxin and antitoxin are proteins, with the antitoxin binding to the toxin and inhibiting its activity [194]. The functions of TAs have long been debated. TAs can play roles ranging from interactions between hosts and their mobile genetic elements, such as viral defense or plasmid stability, to antimicrobial persistence and quorum sensing [194,195]. TA systems are garnering growing attention as potential targets for antibacterial interventions, especially because they are widespread in prokaryotes but lack human analogs. However, the presence and type of TA systems can vary significantly between strains, even within the same species [196]. Targeting TA-based PPIs is also conceptually unique because it can involve either destabilizing the TA complex to unleash the toxin’s lethal activity or stabilizing the complex to prevent toxin-mediated growth arrest and the emergence of antibiotic-tolerant persister cells.

While the examples below focus on the most studied type II TA systems and their potential as molecular targets in Gram-negative strains, many more such systems [195] have only recently been discovered and characterized.

#### 2.5.1. MazEF

**Figure 13:**
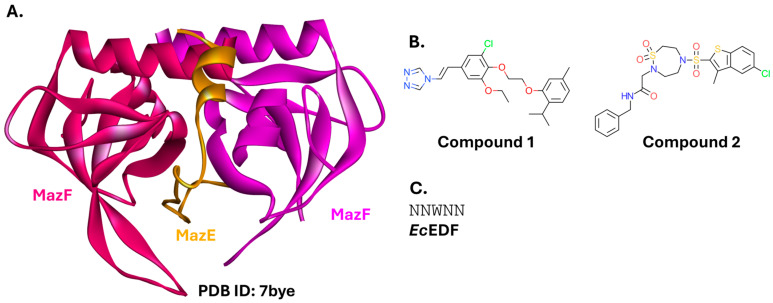
The MazEF complex and its modulators. (**A**). Crystal structure of the *K. pneumoniae* complex (PDB ID: 7bye). (**B**). Structures of compounds targeting the complex. (**C**). Sequence of the *Ec*EDF peptide.

The MazEF system belongs to the type II TA systems and is responsible for bacterial programmed cell death (Figure 13A). The system consists of a stable MazF toxin (an endoribonuclease) and a labile MazE antitoxin (an antidote protein), which form a protein-protein complex under physiological conditions. MazE supply decreases due to stress, e.g., the presence of molecules inhibiting transcription or translation [197], amino acid starvation, DNA damage, or overproduction of the starvation signaling molecule, guanosine tetraphosphate (ppGpp). Consequently, insufficient MazE is present to counteract its constant proteolysis. The pool of unbound MazF increases, and the uninhibited toxin is free to exert its endoribonuclease activity. MazF preferentially hydrolyzes single-stranded RNAs in a sequence-specific manner. The toxin cleaves at ACA sequence sites either at or closely upstream of the start codon of specific mRNAs to generate leaderless mRNAs. MazF also targets 16S rRNA at the decoding center, producing abnormal rRNA lacking the anti-Shine-Dalgarno sequence. This creates an alternative translation machinery responsible for the selective synthesis of specific proteins, some of which, e.g., YfiD, SlyD, ClpX, and YgcR, are involved in cell death [198,199].

The MazE-MazF PPI can be disrupted by Extracellular Death Factors (EDFs). EDFs are short bacterial peptides that can travel from the environment into a bacterial cell and bind to the surface of MazF. This binding prevents the formation of the MazE-MazF complex, releasing the toxic activity of MazF. EDFs can act on multiple bacterial species. Conversely, EDFs of different origins can act on the same strains, as demonstrated by the most studied EDFs targeting *E. coli* [198]. Their activity is well-validated in vitro [198,200], but there is limited research on EDFs as antibacterial agents active in stress-free conditions [201].

Recently, the MazEF TA system was also targeted in *K. pneumoniae* [202]. Using molecular docking, the authors identified two small molecules that bind to MazF, competitively with MazE. Both compounds sustained MazF catalytic activity in the presence of MazE. Compound **1**, containing three separate aromatic rings, exhibited a lower MIC value (63 µM) than Compound **2**, containing a 1,2,5-thiadiazepane 1,1-dioxide moiety (Figure 13B). Antimicrobial activity was evaluated against *K. pneumoniae* ATCC 70021. In the same article, ten peptides derived from the sequences of either MazE or MazF were also investigated. These peptides restored MazF endoribonuclease activity; however, the two peptides tested in the MIC assay were less potent than Compound **1** (MIC = 125 µM) [202]. A group of peptidomimetics based on the EDF peptide sequence from *E. coli* (*Ec*EDF; Figure 13C) was designed using an online tool (pepMMsMIMIC [203]). They docked favorably to *A. baumannii* MazF, compared to the original peptide [204]. However, these findings have not yet been validated experimentally.

#### 2.5.2. VapBC

**Figure 14:**
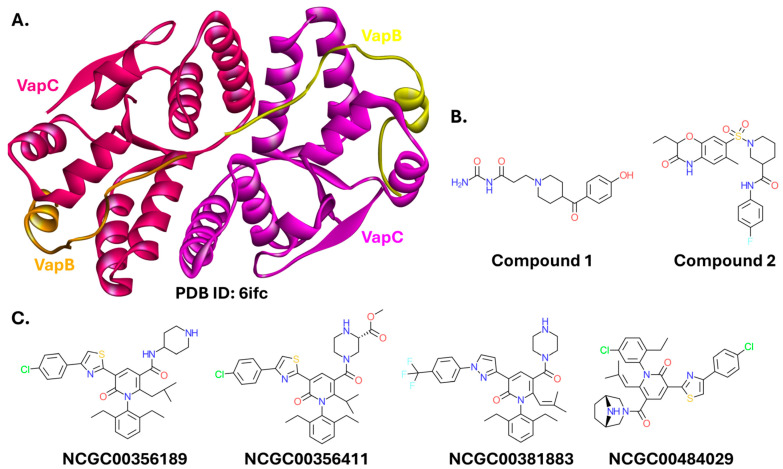
The VapBC complex and its modulators. (**A**). Crystal structure of the *S.* Typhimurium complex (PDB ID: 6ifc) [215]. (**B**). Structures of small molecules targeting the complex, restoring VapC catalytic activity. (**C**). Examples of structures of VapC inhibitors.

In the VapBC TA system (Figure 14A), the VapC toxin is an endoribonuclease, and VapB is the antitoxin. VapC specifically cleaves the initiator RNA, tRNA(fMet), halting translation and leading to cell death. VapB is more labile than VapC; thus, protease activity and stress can shift the VapB-VapC equilibrium, promoting the toxic activity of VapC [205,206,207,208].

VapBC has been most often targeted in *Mycobacterium* strains [209,210,211,212,213]. However, this PPI has also been targeted in Gram-negative bacteria. Kang et al. solved the structure of the VapBC complex from *K. pneumoniae*, and proposed peptide and small-molecule PPI disruptors [214]. The peptides were designed to incorporate VapB and VapC residues crucial for the PPI. The greatest disruption of the VapB-VapC PPI, inferred from the maximal catalytic activity of VapC, was observed with a VapB-derived octapeptide containing a single point mutation (sequence: GMEAQRQL). The authors also performed virtual screening and proposed two small-molecule PPI inhibitors capable of restoring VapC catalytic activity. The compounds have different scaffolds. Compound **1** is an amide with a (4-hydroxyphenyl)(4-piperidinyl)methanone moiety, and Compound **2** is a larger molecule with a 1,4-benzoxazin-3-one ring system (Figure 14B) [214]. However, these compounds were only assayed in vitro.

Sun et al. targeted nontypeable *H. influenzae* (NTHi) [216]. This bacterium causes multiple diseases, including otitis media. Crucially, NTHi is linked to recurrent infections occurring shortly after the completion of antibiotic therapy, indicating that subpopulations of NTHi can survive the antibiotic treatment [217,218], i.e., they are persisters [219]. This phenomenon is associated with one of the two VapBC loci present in the NTHi genome, *vapBC-1* [220]. VapC1 catalytic activity has been linked to the survival of NTHi subpopulations [220,221]. This suggests that inhibiting the VapC1 toxin activity may abolish the induction of NTHi persistence and ultimately prevent recurrent infections. Supported by structural data, the authors screened for small molecules binding to the active site of VapC1, which in the VapB1-VapC1 complex is obstructed by one of the VapB1 helices. They found structurally diverse inhibitors, most often containing a 3-(1,3-thiazol-2-yl)-2(1H)-pyridinone moiety (Figure 14C). The inhibitors were successful in lowering VapC1 activity in a biochemical assay, which presumably and somewhat paradoxically means they disrupt a PPI (direct VapB1-VapC1 interaction) while exerting roughly the same effect as that PPI (abolishing VapC1 catalytic activity). Unfortunately, these compounds did not promote growth in bacterial cells overexpressing VapC1 [216].

#### 2.5.3. PhD-Doc

**Figure 15:**
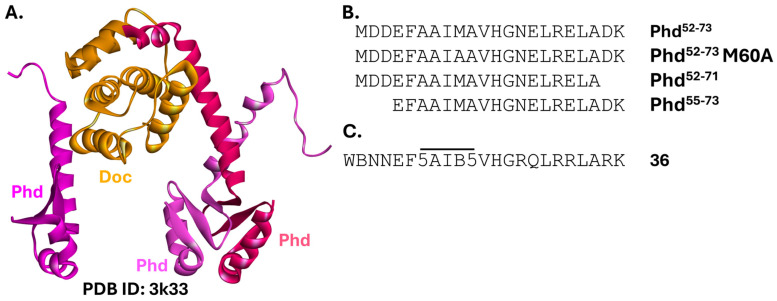
The Phd-Doc complex and its modulators. (**A**). Crystal structure of the complex from a bacteriophage (PDB ID: 3k33) [225]. (**B**). Sequences of peptide inhibitors. (**C**). Sequence of a stapled peptide inhibitor. The black line indicates hydrocarbon stapling. B = l-norleucine; 5 = (*S*)-2-(4-pentenyl)-alanine.

The PhD-Doc type II TA module (Figure 15A) consists of the toxin Doc (death on curing) and the antitoxin Phd (prevents host death). The intrinsically disordered PhD folds into a helical conformation only upon binding to Doc, thereby inactivating it [222,223]. Enzymatically, Doc is a kinase, which, in the absence of Phd, phosphorylates a conserved threonine of the translation elongation factor EF-Tu, preventing it from binding aminoacyl-tRNAs. This, in turn, stalls protein synthesis [224].

The activity of Doc has been associated with an increased number of persister *Salmonella* cells, promoting their survival and reinfection in the host organism [226]. Therefore, the Barnard group formulated a strategy similar to the one targeting VapC1, described in the previous section. Their goal was to design inhibitors of Doc’s catalytic activity to prevent *Salmonella* persister cell formation [227,228]. Peptides derived from the C-terminus of Phd (Figure 15B) inhibited Doc in vitro and when coexpressed with the toxin in *Salmonella* Typhimurium cells [227]. Shortly after, the peptides were optimized through substitutions and hydrocarbon stapling (Figure 15C) to reduce the negative charge of the sequence and improve bacterial uptake, while retaining high affinity for Doc. This yielded peptides efficiently inhibiting Doc both in vitro and in vivo, as well as counteracting the growth inhibition caused by the toxin. The most potent one, Peptide 36, was effective at a 2 µM concentration [228]. To the best of our knowledge, the Phd-Doc TA system has not yet been targeted by small molecules.

#### 2.5.4. HicAB

The HicAB TA system comprises the HicA toxin and the HicB antitoxin. HicAB is genetically unusual because within the *hicAB* operon, the gene encoding the toxin precedes the antitoxin gene, inverting the order typical for most type II TA systems [229]. HicA is a ribonuclease targeting mRNA and transfer-messenger RNA (tmRNA; SsrA). When complexed, HicB covers a large area of HicA, including its active site [230,231,232,233].

HicAB was initially targeted primarily in the Gram-positive *Streptococcus pneumoniae* [233]. However, this TA system is also considered a promising target against Gram-negative strains, including *P. aeruginosa* [234] and *N. gonorrhoeae* [235]. A peptide derived from the HicA helix involved in interactions with HicB (sequence: ELNKYTERGIRKQAG) inhibited the HicA-HicB PPI and increased the catalytic activity of HicA. This peptide inhibited the growth of *S. pneumoniae*. The antimicrobial activity of the peptide was also tested against several Gram-negative strains: *E. coli* ATCC 25922, *Shigella dysenteriae* ATCC 9752, *S.* Typhimurium ATCC 14028, *P. aeruginosa* ATCC 27853, and *K. pneumoniae* ATCC 10031, and the peptide was most potent against the latter strain (MIC = 6.3 µM) [233].

The biological functions of the HicAB TA system are outlined in a recent review [236]. To the best of our knowledge, this TA system has yet to be targeted by small molecules.

#### 2.5.5. HipBA

**Figure 16:**
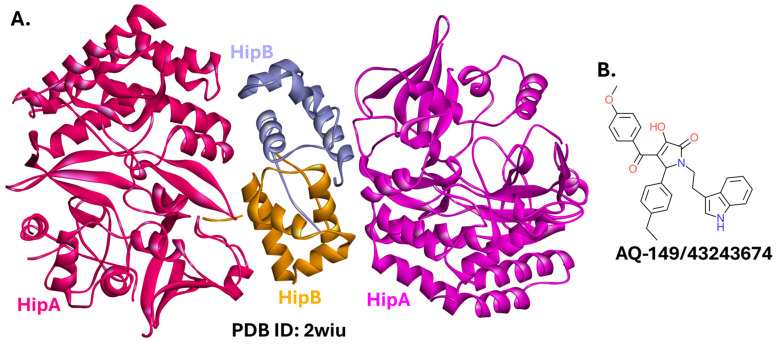
The HipBA complex and its modulators. (**A**). Crystal structure of the complex from *E. coli* (PDB ID: 2wiu) [242]. (**B**). Structure of a small molecule binding to HipA.

In the HipBA TA system (Figure 16A), the HipA toxin is a kinase that phosphorylates glutamate—tRNA ligase (GltX, also called glutamyl-tRNA synthetase), which stalls protein synthesis and induces bacterial cell dormancy and persistence [237,238]. The HipB protein is the system’s antitoxin. Structural studies show that HipB neutralizes HipA not by directly blocking its active site, but by binding to HipA sites distant from the catalytic pocket. HipB inhibits HipA’s kinase activity by locking the HipA toxin into an open, inactive conformation, which prevents the conformational changes required for catalysis [239,240].

The active site of HipA was targeted in a virtual screening campaign. Due to the unusual binding mode of HipB, small molecules that bind the ATP-binding site of HipA are not direct PPI inhibitors. Nevertheless, the promising results obtained by Li et al. are worth highlighting [241]. Compounds from commercially available Specs and Chemdiv libraries bound to HipA and reduced *E. coli* persistence, as measured by cell survival following exposure to an antibiotic. The most effective compound, AQ-149/43243674 (Figure 16B), originating from the SPECS library, exhibited the tightest binding to HipA (K_d_ = 270 nM), and the highest antipersister activity (EC_50_ = 46 µM) [241].

#### 2.5.6. TplE-TplEi

TplE-TplEi is sometimes considered a type II TA system with TplE as the toxin and TplEi serving as the antitoxin. However, it is an unusual system as it is primarily active extracellularly. The Type VI Secretion System (T6SS) recognizes TplE and translocates it into rival, genetically distinct bacterial cells [243,244]. In *P. aeruginosa* infections, TplE has also been shown to induce eukaryotic host cell autophagy by causing disruptions of the endoplasmic reticulum [245]. TplE is a phospholipase that hydrolyzes phospholipids and damages the bacterial cell membrane [245]. The TplEi protein is the system’s antitoxin (in the context of secretion systems, such proteins are sometimes called “immunity proteins” [194]). T6SS is present in Gram-negative bacteria. However, even among the same species, not all express T6SS [246]. Genes encoding T6SSs are found in over 25% of Gram-negative bacterial species [247].

Supported by structural biology studies [248], a peptide mimicking the region of TplE involved in the interaction with TplEi (sequence: DDLFASIGALWTWAWRGPKARQELLKA) was designed. This peptide binds to TplEi and inhibits the growth of *E. coli* BL21 (DE3) cells overexpressing the system and the plasmid-encoded peptide [249]. To the best of our knowledge, the TplE-TplEi PPI has not been modulated by small molecules.

Protein–protein interaction systems and their modulators described in this review are summarized below (Table 1).

## 3. Future Perspectives

The field of drug discovery aimed at PPIs, which was non-existent until relatively recently, is developing rapidly. This is due to progress in key areas.

First, obtaining reliable structural data, which is crucial for the rational design of PPI modulators, is now easier than ever. Modern protein crystallization laboratories and synchrotron facilities employ extensive automation [250,251,252,253,254,255], drastically increasing throughput and accessibility to non-experts. Cryo-electron microscopy (cryoEM) also remains an indispensable technique for structural biologists [256], with dedicated solutions to issues that may arise when solving structures of protein-protein complexes [257,258,259], as is the case with Nuclear Magnetic Resonance (NMR) spectroscopy [260,261]. Microcrystal electron diffraction (microED) is undergoing rapid development and will likely join other structural biology techniques routinely used for studying PPIs [262].

Arguably, the most significant recent breakthrough in all structure-based research stems from the rapid rise of artificial intelligence tools, most notably since the release of AlphaFold2 in 2021 [263]. Computational protein structure prediction has become far more reliable. Although AlphaFold2 was initially designed to model only single-chain proteins, methods to model protein-peptide, protein-protein, and multiprotein complexes quickly emerged [264,265,266,267,268,269,270]. However, AlphaFold3 overcomes these limitations out of the box and can model not only protein complexes but also their interactions with nucleic acids, small molecules, or ions [271]. Other predictive software solutions are also emerging [272,273,274].

Second, the rational targeting of PPIs, which relies on identifying hotspot residues, benefits from numerous methods. Apart from the classic experimental technique of alanine scanning mutagenesis [275], several computational methods for identifying hotspot residues are available. These include calculating per-residue binding energies in molecular dynamics trajectories [276,277,278], in silico alanine scanning [279,280,281], and multiple proprietary tools [282,283,284,285,286,287].

Third, recent developments in virtual screening techniques have become applicable to PPI modulator discovery, including the capacity to explore extremely large sets of chemicals. Virtual screening relies on molecular docking and scoring algorithms to identify molecules with the most favorable predicted binding energy to the selected target. These molecules are considered the most promising candidates for potent modulators of the target PPI. Larger virtual screening libraries increase the probability of finding promising hits. However, even powerful modern computer hardware struggles to screen the largest chemical libraries in their entirety. Chemical libraries composed of building blocks that can be combined using a limited number of reaction protocols are extremely massive. These are often called “chemical spaces”, and instead of containing full molecular structures, they include only the building blocks with rules on how to chemically combine them. For example, KnowledgeSpace [288] released by BioSolveIT GmbH contains 10^15^ compounds, and the library from GlaxoSmithKline [289] has 10^26^ compounds. Although these libraries would be intractable using classic docking algorithms, even when highly parallelized, newly developed techniques enable exploration of these vast chemical spaces. One such approach uses predictive machine learning-based models to derive docking scores, reducing the computational cost by over 1000-fold compared to standard docking protocols. A classification algorithm is trained on a set of molecular docking results for the target protein, and is then used to make selections from compounds in a massive library [290]. In a similar, artificial intelligence-driven solution, screening speeds are estimated to be 50 times faster than conventional docking [291,292]. There are also hierarchical approaches that do not require machine learning. They rely on estimating the affinities of the building fragments present in the libraries and then, guided by the chemical rules governing their connectivity, computationally assemble larger molecules containing the initial fragments [293,294].

Overall, computational techniques are indispensable to modern drug discovery studies. Virtual screening enables rapid, cost-effective selection of compounds for further investigation. This selection can be further refined by testing the stability of protein-ligand complexes in molecular dynamics simulations and estimating the free energy of binding of a ligand. This approach allows researchers to validate experimentally only the most promising compounds, while retaining the ability to initially screen massive compound libraries. This workflow was commonly used in the studies described in this review.

Finally, many experimental techniques for studying PPIs and their modulators are either well-established or are being developed to increase throughput, improve ease of use, and promote wider use within laboratories. They have been recently succinctly reviewed [295,296]. Other, equally in-depth reviews provide an overview of the entire process of identifying PPIs and designing their modulators [297,298].

The number of PPI complexes, i.e., the size of the protein-protein interactome, in bacteria is difficult to measure accurately. Undoubtedly, there are a vast number of such interactions, with their number potentially exceeding tens of thousands in *E. coli* [299,300]. This means that many promising PPI targets in Gram-negative bacteria likely remain understudied, poorly understood, or even unknown. However, even the list of targetable PPIs presented in this review does not exhaust all known targets with either already known modulators or active research underway. As this review focuses on PPIs with significant recent developments, some complexes were omitted. These include, among others: the assembly of T6SS [301], the Lol complex responsible for the transport of lipoproteins to the outer membrane [302,303,304], and several type II TA systems such as ε_2_ζ_2_ [305,306,307], HigBA [308,309,310], and RelBE [311,312,313]. Potential targets also extend beyond the type II TA systems. For instance, type IV TA systems, in which the toxin and antitoxin, instead of interacting directly, compete for another cellular target, represent interesting targets for PPI modulators. In one such system, CptBA-like, the CptA toxin inhibits the polymerization of cytoskeletal proteins, whereas the antitoxin, CptB, binds to these proteins, stabilizing them [314].

PPI modulators can exert diverse effects on their target systems, resulting in distinct biochemical and biological consequences. PPI inhibitors can stall the biological process governed by the interaction, as is the case with RNAP core-σ^70^ PPI inhibitors (e.g., [171]), or conversely, activate a toxic function as seen with TA system inhibitors that restore the toxin’s activity ([233] and others). In type II TA systems, PPI inhibitors restoring the toxin’s activity can bind either to the antitoxin or to the toxin at an allosteric site, as is the case with EDFs binding on the surface of MazF [201]. However, a TA system might also be targeted to inactivate the toxin to combat bacterial persistence. This could be achieved with PPI disruptors binding to the toxin’s active site competitively with the antitoxin, as with small molecules targeting NTHi [216], but also through other means, such as stabilizing the TA PPI. Ongoing research and future developments are expected to yield additional PPI targets, facilitating more sophisticated strategies for PPI modulation.

## 4. Conclusions

As the consequences of increasing AMR become ever more imminent, novel strategies for antibacterial drug discovery are garnering attention. Modulators of PPIs are a prime example of such a strategy. This approach is especially promising because it may slow the development of resistance. A drug targeting a single enzyme might lose its efficacy after a single mutation in its molecular target. However, for most PPI modulators, resistance may necessitate simultaneous mutations in both protein partners for the drug to lose its efficacy, while allowing the protein-protein complex to remain stable.

Gram-negative bacteria are pathogens of critical importance, as evidenced by the WHO Bacterial Priority Pathogens List [10]. Their cell envelope forms a permeation barrier for many promising drug candidates. Compounds aimed at PPIs located within the outer membrane or the periplasm should therefore reach their targets more easily. However, even cytosolic targets could be accessed, perhaps through chemical conjugation of molecules, an approach that has been proven effective when applied to several bacterial targets, not necessarily PPIs [315,316,317,318,319,320,321,322,323,324,325,326,327,328,329].

PPIs are natural targets for peptides or peptidomimetics derived from the sequence of one of the interaction partners. Often, hotspot residue clusters also enable small-molecule modulation. However, large and flat interfaces remain intractable for such compounds. Since peptides are usually less attractive drug candidates than small molecules due to their susceptibility to proteolysis, limited permeability, and poor oral bioavailability, PPIs that cannot be disrupted by small molecules are considered less promising molecular targets. RcsF-IgaA, FimC-FimH, Phd-Doc, HicAB, and TplE-TplEi complexes were targeted solely by peptides and peptidomimetics. Nonetheless, this might not be indicative of the structural features of these PPI interfaces but rather reflects the limited research that went into each target. Future studies may very well identify their ortho- or allosteric small-molecule modulators.

Overall, many developments in recent decades have made PPIs not only druggable but often attractive molecular targets. This statement holds true not only for anticancer and antiviral chemotherapy but also for targeting bacteria, including Gram-negative strains. Bacterial PPIs are commonly distinct from eukaryotic interactions, providing ample opportunities for selective targeting. The upcoming years are expected to bring new discoveries and an enhanced understanding of pathogen-pathogen PPIs in Gram-negative bacteria, which, hopefully, will help alleviate the severe burden of antibiotic resistance.

## Figures and Tables

**Table 1:** Summary of protein–protein interactions and their modulators described in this review.

PPI Complex	Molecular Target	Compound Type	Remarks	References
BAM complex	Within BAM complex	Small molecule	Exact mechanism unclear	[37]
				[38]
	BamA	Small molecule		[39]
	Within BAM complex	Small molecule	Inhibition depends on BamA, BamB, BamD, and BamE	[40]
	BamD	Peptide		[41]
	BamA	Peptide	Binds at the BamA barrel domain	[42,43,44]
				[45]
Rcs	IgaA	Peptide	BamA as a secondary target	[60]
Lpt	LptA	Peptide		[67,68,69]
		Small molecule		[70]
	LptA and LptC	Small molecule		[71]
				[72]
	LptD	Peptidomimetic	In clinical trials	[73,74,75]
	LptF	Peptidomimetic	In clinical trials	[78,79]
FimC-FimH	FimH	Peptide		[91]
FtsZ-ZipA	ZipA	Small molecule	Toxic to eukaryotic cells	[97,98]
				[99]
FtsZ oligomer	FtsZ	Small molecule		[101]
			Toxic to eukaryotic cells	[102]
				[105]
				[106]
				[107]
				[110]
				[111]
				[112]
		Peptide		[113]
				[114]
		Modified peptide		[115]
SSB and partner proteins	ExoI	Small molecule		[130]
		Peptide		[132]
		Modified peptide		[133]
	RecO	Modified peptide		[133]
	PriA	Small molecule	Structures not disclosed	[137]
				[138]
				[130]
	DnaG	Fragment/small molecule		[140]
β-sliding clamp and partner proteins	β-sliding clamp	Small molecule		[148]
		Small molecule and modified peptide		[149]
		Fragment		[150]
		Small molecule		[151]
				[152]
		Peptides		[153]
		Modified peptide		[154]
			Covalent inhibitors	[155]
				[157]
RNAP core-σ^70^	Within the complex	Small molecule	Effective on *E. coli* with efflux system deficiency	[165]
	β’	Small molecule		[166]
	β’ or σ factor	Small molecule	Improved permeability	[167]
	β’	Small molecule		[168]
			Require cell permeabilizer	[169]
				[170]
		Peptide	Do not affect bacterial growth	[171]
		Modified peptide	Loss of inhibitory activity	[172]
RNAP-NusG	β’	Small molecule		[173]
NusB-NusE	NusB	Small molecule		[180]
		Peptide		
		Small molecule		[181]
			Nusbiarylins	[183]
				[184]
				[185]
				[187]
L10-L12	L10 and L12	Small molecule	Capable of binding to either PPI partner	[192]
MazE-MazF	MazF	Peptide	Effective only under stress conditions	[198,200,201]
		Small molecule		[202]
		Peptide		[202]
		Modified peptide		[204]
VapB-VapC	VapC	Peptide		[214]
		Small molecule		[214]
VapB1-VapC1	VapC1	Small molecule	Toxin inhibitor to combat persisters	[216]
PhD-Doc	Doc	Peptide	Toxin inhibitor to combat persisters	[227]
		Modified peptide		[228]
HicA-HicB	HicB	Peptide		[233]
HipB-HipA	HipA	Small molecule	Not targeting PPI directly. Toxin inhibitor to combat persisters	[241]
TplE-TplEi	TplEi	Peptide		[249]

## Data Availability

No new data were created or analyzed in this study. Data sharing is not applicable to this article.
